# Associations of Plasma Selenium with Arsenic and Genomic Methylation of Leukocyte DNA in Bangladesh

**DOI:** 10.1289/ehp.1001937

**Published:** 2010-09-15

**Authors:** J. Richard Pilsner, Megan N. Hall, Xinhua Liu, Habibul Ahsan, Vesna Ilievski, Vesna Slavkovich, Diane Levy, Pam Factor-Litvak, Joseph H. Graziano, Mary V. Gamble

**Affiliations:** 1 Department of Environmental Health Sciences; 2 Department of Epidemiology and; 3 Department of Biostatistics, Mailman School of Public Health, Columbia University, New York, New York, USA; 4 Department of Health Studies; 5 Department of Medicine and; 6 Human Genetics and Cancer Research Center, University of Chicago, Chicago, Illinois, USA; 7 Department of Pharmacology, College of Physicians and Surgeons, Columbia University, New York, New York, USA

**Keywords:** arsenic, Bangladesh, DNA methylation, epigenetics, folate, folic acid, selenite, selenium, thioredoxin reductase, well water

## Abstract

**Background:**

Global hypomethylation of DNA is thought to constitute an early event in some cancers and occurs in response to arsenic (As) exposure and/or selenium (Se) deficiency in both *in vitro* and animal models. In addition, antagonism between As and Se, whereby each reduces toxicity of the other, has been well documented in animal models. Se status may therefore modify the health effects of As in As-exposed populations.

**Objective:**

The primary objectives of our study were to test the hypothesis that Se deficiency is associated with genomic hypomethylation of lymphocyte DNA and to determine whether Se levels are associated with blood As (bAs) and urinary As (uAs) concentrations in adults exposed to As-contaminated groundwater in Bangladesh. A secondary objective was to explore the relationships between plasma Se and As metabolites.

**Design:**

We assessed plasma Se concentrations, As metabolite profiles in blood and urine, and genomic methylation of leukocyte DNA in a cross-sectional study of 287 adults.

**Results:**

After adjustment for potential confounders, we observed an inverse association between Se (micrograms per liter) and genomic DNA methylation (disintegrations per minute per 1-μg/L increase in Se): β = 345.6; 95% confidence interval (CI), 59–632. Se concentrations were inversely associated with total As concentrations (micrograms per liter) in blood (β = −0.04; 95% CI, −0.08 to −0.01) and urine (β = −20.1; 95% CI, −29.3 to −10.9). Se levels were negatively associated with the percentage of monomethylarsinic acid (β = −0.59; 95% CI, −1.04 to −0.13) and positively associated with the percentage of dimethylarsinic acid (β = 0.53; 95% CI, 0.04 to 1.01) in blood.

**Conclusions:**

Our results suggest that Se is inversely associated with genomic DNA methylation. The underlying mechanisms and implications of this observation are unclear and warrant further investigation. In addition, Se may influence bAs and uAs concentrations, as well as relative proportions of As metabolites in blood.

Arsenic (As) and selenium (Se) share many chemical properties and are adjacent on the periodic table of elements. The two metalloids, however, have marked differences in their biological effects (Csanaky and Gregus 2003). Although toxic at high doses, Se is an essential trace element necessary for antioxidant enzyme activity, thyroid hormone metabolism, and immune function and has been used in chemoprevention studies (Zeng et al. 2005). In contrast, As has no known biological function and displays both acute and chronic toxicity. Arsenic-contaminated groundwater is a major health concern worldwide, affecting roughly 140 million people in > 70 countries (Kinniburgh and Smedley 2001; World Bank 2005). Ingestion of inorganic As (InAs) via contaminated drinking water is associated with elevated risk of premalignant skin lesions and cancers of the skin, lung, bladder, liver, and kidney, as well as noncarcinogenic outcomes, including cardiovascular disease and neurological deficits (National Research Council 2001; Tseng 2008; Wasserman et al. 2004).

In drinking water, As occurs in its inorganic form, either as arsenite (As^III^) or arsenate (As^V^), the former being the primary form found in groundwater in Bangladesh. Once ingested, As^III^ undergoes oxidative methylation using *S-*adenosylmethionine (SAM) as the methyl donor, forming monomethylarsonic acid (MMA^V^). MMA^V^ can then be reduced to monomethylarsonous acid (MMA^III^), with reducing equivalents provided by thioredoxin (Trx) (Thomas et al. 2004). MMA^III^ can undergo a second methylation step to form dimethylarsinic acid (DMA^V^), the major metabolite found in urine. Population studies have shown that individuals having a relatively lower capacity to methylate As to DMA^V^ are at greater risk for skin cancers (Chen et al. 2003; Hsueh et al. 1997; Yu et al. 2000), bladder cancer (Huang et al. 2008), and peripheral vascular disease (Tseng et al. 2005).

Unlike As, where contaminated drinking water is the predominant route of high exposure, most Se in Bangladesh is obtained from the diet; the concentrations of Se in drinking water in Bangladesh are well below the World Health Organization (WHO) guideline of 10 μg/L (Frisbie et al. 2009). Dietary Se, primarily in the form of selenomethionine, undergoes hepatic transsulfuration, generating free selenocysteine and metabolically active Se. The latter can be incorporated into selenoproteins, including selenoprotein P (a Se-rich plasma protein involved in Se transport), glutathione peroxidases, Trx reductase (TR), and other selenoproteins (Burk et al. 2006). A second pathway results in the production of selenosugars that are excreted in urine (Kobayashi et al. 2002; Kuehnelt et al. 2007). Although, like As, some Se can be metabolized by serial methylation reactions (Birringer et al. 2002; Ohta et al. 2009), recent evidence indicates that this pathway is likely relatively minor (Kuehnelt et al. 2007).

Antagonistic effects between As and Se have been well documented since the original discovery by Moxon (1938) that cattle could be protected from Se toxicity by treatment with As. Several studies have indicated that As and Se each mutually facilitate excretion of the other in bile (Levander 1977). Recent work in rabbits indicates that this may occur via the formation of a Se–As–glutathione conjugate (Gailer et al. 2000), although this has not yet been demonstrated in humans. Other proposed mechanisms include direct interaction and precipitation of As and Se in renal cells and effects on cellular signaling, zinc finger proteins, and methylation pathways (Berry and Galle 1994; Zeng et al. 2005).

The biological interactions between As and Se evoke the possibility that Se may influence the development of As-induced health outcomes (Gailer 2009). A recent case–control study from our Bangladeshi cohort found that blood Se concentrations were inversely associated with total urinary As (uAs) concentrations and with the risk of As-related premalignant skin lesions (Chen et al. 2007). However, not all of the available evidence supports a beneficial effect of Se on As metabolism and toxicity (Geng et al. 2009; Kenyon et al. 1997; Styblo and Thomas 2001; Zakharyan et al. 1995). The effects of dietary Se (i.e., selenomethionine) on As metabolism have not previously been investigated.

Several studies have investigated the impact of Se on epigenetic processes. DNA methylation, which also requires SAM as the methyl donor, involves the covalent addition of a methyl group to the 5-carbon of cytosine bases located within CpG dinucleotides. It is an essential epigenetic modification that influences gene expression, X chromosome inactivation, and silencing of endogenous retroviruses. In animal models, dietary deficiency of Se has been reported to cause genomic hypomethylation of liver and colon DNA (Davis et al. 2000), whereas supplementation with sodium selenite (Se^IV^) increased DNA methyltransferase (DNMT) activity and genomic DNA methylation (Davis and Uthus 2003). Conversely, *in vitro* studies in prostate cancer cells revealed that Se^IV^ treatment led to decreased genomic DNA methylation (Xiang et al. 2008).

The primary objectives of this study were to test the hypothesis that Se deficiency is associated with genomic hypomethylation of lymphocyte DNA and to determine whether Se levels were associated with blood As (bAs) and uAs concentrations in adults exposed to As-contaminated groundwater in Bangladesh. A secondary objective was to explore the relationships between Se and As metabolites.

## Materials and Methods

The Nutritional Influences on Arsenic Toxicity (NIAT) study was designed to assess the prevalence of folate deficiency and hyperhomocysteinemia in Bangladesh (Gamble et al. 2005a), to determine whether folate nutritional status is associated with methylation of As (Gamble et al. 2005b), and to determine if folate deficiency and/or As exposure is associated with genomic methylation of leukocyte DNA (Pilsner et al. 2007). The NIAT study was conducted in collaboration with the Health Effects of Arsenic Longitudinal Study (HEALS), a large prospective cohort study of adults exposed to a wide range of water As concentrations, from which the present participants were selected (Ahsan et al. 2006).

### The region

The study site is a 25-km^2^ region within Araihazar, approximately 30 km east of Dhaka, Bangladesh. The variability of well-water As concentrations (0.1–860 μg/L) in this region offers a unique opportunity to study dose–response relationships between As exposure and As-related health outcomes. Our data on socioeconomic status, and data from Columbia University’s Center for International Earth Science Information Network (2007), indicate that this region is not particularly poor by Bangladesh standards.

### Eligibility criteria and study design

The HEALS cohort study originally included 11,746 men and women between 18 and 65 years of age who were recruited between October 2000 and May 2002 and who continue to be followed at 2-year intervals. The cohort has since been expanded to include nearly 20,000 participants. As part of the NIAT study, a cross-sectional study of 1,650 of the original participants (Gamble et al. 2005a) was first conducted to determine the prevalence of folate and cobalamin (B_12_) deficiencies and of hyperhomocysteinemia, and to identify a pool of participants with low plasma folate for recruitment into a folic acid intervention study. The 200 participants enrolled in the folic acid supplementation trial were a random sample of the 550 participants who fell into the lowest tertile of plasma folate from the cross-sectional study (Gamble et al. 2005a). Participants were excluded if they were pregnant, B_12_ deficient (B_12_ ≤ 185 pmol/L), or were taking vitamin supplements.

For these analyses, we have included baseline data from 195 participants who completed the folic acid intervention study from whom high-quality DNA was obtained (Gamble et al. 2006, 2007). An additional 100 participants were recruited from the HEALS cohort study in order to capture a wider range of As exposure and folate nutritional status. These participants were known to have continued to drink from the same well for at least the past 4 years; we oversampled participants who were drinking from a high-As well such that 70% were drinking water with > 50 μg As/L. Aside from these criteria, these 100 participants were randomly selected. After excluding participants with missing values for plasma Se or folate and those with insufficient DNA for the measurement of genomic DNA methylation, 287 Bangladeshi adults were included in the present study. Oral informed consent was obtained by our Bangladeshi field staff physicians, who read an approved assent form to the study participants. This study was approved by the institutional review boards of Columbia Presbyterian Medical Center and the Bangladesh Medical Research Council.

### Analytic techniques

#### Sample collection and handling

Blood samples for buffy coats, plasma total homocysteine, folate, and total B_12_ were obtained by venipuncture at the time of recruitment. Blood was collected into heparin-containing Vacutainer tubes, which were placed in IsoRack/IsoPack cool packs (Brinkmann Instruments, Westbury, NY) designed to maintain samples at 0°C for 6 hr. Within 4 hr, samples were transported in hand-carried coolers to our local laboratory, situated in our field clinic in Araihazar. Samples were centrifuged at 3,000 × *g* for 10 min at 4°C, and buffy coat and plasma were separated from red cells. Aliquots of plasma were stored at −80°C and shipped frozen on dry ice to Columbia University for analysis. Urine samples were collected in 50-mL acid-washed polypropylene tubes. These were kept in portable coolers, frozen at −20°C within 4 hr, and shipped on dry ice.

#### Water As

A survey of all wells in the study region assessed water As concentrations of tube wells at each participant’s home between January and May 2000 (Van Geen et al. 2002). Samples were analyzed at Columbia University’s Lamont Doherty Earth Observatory by graphite furnace atomic absorption (GFAA), which has a detection limit of 5 μg/L. Those samples found to have nondetectable As concentrations by GFAA were subsequently analyzed by inductively coupled mass spectrometry (ICP-MS), which has a detection limit of 0.1 μg/L (Cheng et al. 2004).

#### uAs metabolites and total uAs

Total uAs concentrations were measured by GFAA spectrometry using an Analyst 600 graphite furnace system (PerkinElmer, Shelton, CT) in the Columbia University Trace Metals Core Lab, as described previously (Nixon et al. 1991). Our laboratory participates in a quality-control program for total uAs coordinated by P. Weber at the Quebec Toxicology Center (Quebec, Canada). During the course of this study, intraclass correlation coefficients between our laboratory’s values and samples calibrated at Weber’s laboratory were 0.99. Urinary creatinine was analyzed using a method based on the Jaffe reaction (Slot 1965) and was used to correct for hydration status. Arsenic metabolites were speciated using HPLC separation of arsenobetaine (AsB), arsenocholine (AsC), As^V^, As^III^, MMA (MMA^III^ + MMA^V^), and DMA (DMA^V^), followed by detection using ICP-MS. We calculated the percentages of InAs (InAs^III+V^; %InAs), MMA (%MMA), and DMA (%DMA) after subtracting AsC and AsB from the total. The limit of detection for each uAs metabolite was 0.1 μg/L. Values below the limit of detection were treated as zero. The interassay coefficients of variation (CVs) were 5.8% for total As, 7.8% for As^III^, 18.3% for As^V^, 5.8% for MMA, and 3.1% for DMA. The intraassay CVs were 3.6% for total As, 4.2% for As^III^, 10.9% for As^V^, 2.3% for MMA, and 1.4% for DMA.

#### Plasma Se

Plasma samples were analyzed for Se using an Elan Dynamic Reaction Cell (DRC) II ICP-MS equipped with an AS 93+ autosampler (PerkinElmer). The ICP-MS-DRC method for metals in plasma was developed according to published procedures (Pruszkowski et al. 1998), with modifications for plasma sample preparation developed in our laboratory (Chen et al. 2007). Although there are no well-established guidelines defining Se deficiency, for the purposes of this study we chose to define Se deficiency as plasma Se < 70 μg/L. This cutoff is based on reports that supplementation with increasing doses of selenomethionine leads to increases in the concentration and activity of glutathione peroxidases until the dose–response relationship reaches a plateau at plasma Se levels between 70 and 90 μg/L (Food and Nutrition Board 2000; Xia et al. 2005).

#### bAs metabolites and total bAs

A subset of 223 subjects had bAs metabolite data available from a previous study (Gamble et al. 2007). This subset represented individuals with higher As exposure in which almost all blood metabolite measurements were above the limit of detection of 0.1 μg/L. The interassay CVs were 6.1% for total bAs, 7.5% for As^III^, 11.6% for As^V^, 5.8% for MMA, and 5.0% for DMA. The intraassay CVs were 4.0% for total bAs, 2.6% for As^III^, 7.7% for As^V^, 6.2% for MMA, and 5.5% for DMA. bAs metabolites and total bAs were measured as previously described (Gamble et al. 2007; Hall et al. 2006; Pilsner et al. 2007).

#### Isolation of leukocyte DNA

Buffy coats were transferred to 1 mL RBC Lysis Solution (GenomicPrep Blood DNA Isolation Kit, catalog no. 27-5236-01; Amersham Biosciences, Piscataway, NJ) and then centrifuged at 16,000 × *g* for 5 min to separate leukocytes from contaminating red blood cells. Leukocytes were subsequently lysed in the presence of a DNA preservative and stored at 4°C. Samples were then shipped at 4°C to Columbia University, where the isolation of leukocyte DNA was completed following the manufacturer’s protocol.

#### Genomic DNA methylation

Genomic methylation of leukocyte DNA was determined in 500 ng DNA using the methyl acceptance assay method of Balaghi and Wagner (1993). DNA was incubated with [^3^H]SAM in the presence of the SssI prokaryotic methylase enzyme, which indiscriminately methylates all unmethylated CpG sequences. Therefore, the ability of DNA to incorporate [^3^H]methyl groups is inversely related to endogenous DNA methylation. Briefly, 0.5 μg DNA was incubated with 3 U SssI methylase (New England Biolabs, Beverly, MA), 3.8 μM (1.1 μCi) ^3^H-labeled SAM (PerkinElmer), EDTA, DTT (dithiothreitol), and Tris-HCl (pH 8.2) in a 30-μL mixture and incubated for 1 hr at 37°C. The reaction was terminated on ice, and 15 μL of the reaction mixture was applied onto Whatman DE81 filter paper. The filter was washed on a vacuum filtration apparatus three times with 5 mL 0.5 M sodium phosphate buffer (pH 8.0), followed by 2 mL each of 70% and 100% ethanol. Dried filters were each placed in a vial with 5 mL scintillation fluid (Scintisafe; Fisher, Fair Lawn, NJ) and were analyzed by a Tri-Carb 2100TR Liquid Scintillation Analyzer (Packard Instruments, Downers Grove, IL). Each DNA sample was processed in duplicate, and each processing run included samples for background (reaction mixture with all components except SssI enzyme), a hypomethylation control (HeLa cell DNA), and a quality-control sample (DNA extracted from a whole-blood sample) to determine the inter- and intraassay CVs (1.8% and 5.3%, respectively). To quantify the amount of double-stranded DNA (dsDNA) in each reaction, an aliquot of the assayed DNA was used to determine DNA concentrations using PicoGreen dsDNA Quantitation Reagent (Molecular Probes, Eugene, OR). All disintegrations per minute (dpm) values were expressed per microgram of DNA as quantified by PicoGreen.

#### Plasma folate and B_12_

Plasma folate and total B_12_ were analyzed by radioimmunoassay (Quantaphase II; Bio-Rad Laboratories, Richmond CA) as previously reported (Gamble et al. 2005a). The intra- and interassay CVs for folate were 3% and 11%, respectively, and those for B_12_ were 4% and 8%, respectively.

### Statistical methods

We calculated descriptive statistics for general characteristics as well as for plasma nutrients and uAs metabolite concentrations. Paired *t*-tests were used to detect differences between As metabolites in blood and urine. Bivariate associations between plasma Se and covariates and between Se and As variables were examined using Spearman correlation coefficients. To further examine the associations between plasma Se and the outcomes of interest, after adjustment for potential confounders, we employed linear regression models with the main predictor, plasma Se, as a continuous variable. We identified potential confounders as variables that were known to be associated with the outcomes of interest and that were also associated with plasma Se in this study sample. These variables included age, body mass index (BMI), betel nut use, plasma folate, plasma B_12_, urinary creatinine, and water As. We also adjusted for sex and smoking, although these variables were not associated with plasma Se. All potential confounders were modeled as continuous variables except sex and the variables cigarette smoking and betel nut use (both modeled as ever/never use). Variables with skewed distributions were log or square root transformed before inclusion in linear regression models to create approximately normal distributions for dependent variables, to improve the linearity of the relationship between independent and dependent variables, or to reduce the impact of extreme values of an independent variable. Plasma folate and B_12_, urinary creatinine, and total bAs were log transformed, whereas water As was square root transformed.

For the outcome variables in the linear regression models that were significantly associated with plasma Se as a continuous predictor, we then applied linear regression models with categorized Se to describe specific patterns of the associations. We created five categories of plasma Se and computed covariate-adjusted mean values of the outcome variables for each category. All of the Se-deficient participants were included in the first category; the Se-sufficient participants were distributed equally among the remaining categories (quartiles).

All analyses were performed using SAS (version 9.1; SAS Institute Inc., Cary, NC); all statistical tests were two sided with a significance level of 0.05.

## Results

The characteristics of the study population are presented in Table 1. The mean age was 37.9 years; approximately half of the study population was female. The average BMI was 19.8, and 41.7% of participants had a BMI < 18.5, reflecting the high prevalence of underweight in this population (Centers for Disease Control and Prevention 2007). Our data show that 89% of participant’s homes had corrugated tin roofs, indicative of moderate socioeconomic status. Mean plasma Se was 87.6 μg/L, and we considered 16% of participants Se deficient based on a cutoff value of 70 μg/L.

Water As concentrations ranged from 0.1 to 716 μg/L, with 66% of wells exceeding the Bangladeshi standard of 50 μg/L and 84% of wells exceeding the WHO standard of 10 μg/L. [^3^H]Methyl incorporation (genomic DNA methylation) ranged from 27,256 to 56,940 dpm/μg DNA. In a subset of individuals (*n* = 223) for whom bAs data were available, the mean bAs concentration was 9.9 μg/L. We found marked differences in mean proportions of As metabolites in blood versus urine (DMA, 33.4% vs. 72.4%; MMA, 40.3% vs. 12.6%; InAs, 26.3% vs. 15.1%; paired *t*-test, *p* < 0.0001 for all metabolites), consistent with the fact that DMA is preferentially excreted in urine.

In the linear regression model presented in Table 2, the estimates represent the change in mean levels of each outcome variable associated with a 1-μg/L increase in plasma Se with and without adjusting for age, sex, BMI, smoking, betel nut use, plasma folate, plasma B_12_, urinary creatinine, and water As. Plasma Se was positively correlated with [^3^H]methyl incorporation. Because [^3^H]methyl incorporation is inversely related to genomic DNA methylation, this observation indicates that plasma Se concentrations are inversely related to genomic DNA methylation. Although we have previously shown that As exposure is associated with increased genomic DNA methylation (Pilsner et al. 2007), in the present analyses the association between plasma Se and [^3^H]methyl incorporation was unaltered by the inclusion of water As in the regression models.

Plasma Se was inversely associated with total uAs with and without adjusting for covariates (Table 2). We found no significant associations between plasma Se and the percent distribution of uAs metabolites. Increasing plasma Se was also associated with a decreasing level of total bAs in unadjusted and adjusted linear regression models. In addition, plasma Se was inversely associated with blood %MMA and positively associated with blood %DMA. We found no association between plasma Se and blood %InAs.

Adjusted mean values of [^3^H]methyl incorporation and As variables by category of plasma Se are presented in Table 3. [^3^H]Methyl incorporation into genomic DNA generally increased with increasing plasma Se categories, although there appeared to be little difference between the second and third categories. Adjusted mean values of total uAs decreased with increasing categories of plasma Se. For total bAs and blood %MMA, there appeared to be a threshold effect; adjusted mean values in the first three categories of plasma Se were similar and higher than the values in the two highest categories. There also appeared to be a threshold in the association between plasma Se and blood %DMA such that the adjusted mean values in the three lowest categories of plasma Se were similar and lower than the values in the two highest categories.

## Discussion

In this cross-sectional study of 287 Bangladeshi adults, we observed that plasma Se is inversely associated with genomic methylation of leukocyte DNA, with and without adjustment for covariates including plasma folate concentrations and water As. In addition, we found inverse associations between plasma Se and both total uAs and bAs concentrations. Plasma Se was also inversely associated with %MMA and positively associated with %DMA in blood.

### Plasma Se and genomic DNA methylation

Our findings, which suggest that plasma Se concentrations are inversely related to genomic DNA methylation, are contrary to our original hypothesis that Se deficiency would be associated with genomic hypomethylation of DNA. Previous animal and *in vitro* studies examining the influence of Se on DNA methylation have been inconsistent. A series of animal studies indicate that dietary Se deficiency caused genomic hypomethylation of liver and colon DNA (Davis et al. 2000; Uthus et al. 2006), whereas supplementation with Se^IV^ increased DNMT activity and DNA methylation in the liver and colon, respectively (Davis and Uthus 2003). *In vitro* studies using Friend erythroleukemic and HCT116 colon carcinoma cells, however, have shown that Se^IV^ exposure caused a decrease in DNMT activity concomitant with DNA hypomethylation (Cox and Goorha 1986; Fiala et al. 1998). Additional data indicate that Se^IV^ treatment induced genomic DNA hypomethylation and was associated with the down-regulation of DNMT 1 and DNMT 3a expression and histone deacetylase activity in the LNCaP human prostate cancer cell line (Xiang et al. 2008). Although speculative, our results are consistent with the *in vitro* assays, suggesting that the inverse association between plasma Se concentrations and genomic DNA methylation in our study could reflect a decrease in DNMT expression and/or activity.

### Plasma Se and total As concentrations

Previous work has suggested that the association between As exposure and clinical outcomes may be modified by Se nutritional status (Verret et al. 2005). This hypothesis is supported by experimental studies indicating that As and Se each mutually facilitate biliary excretion of the other (Zeng et al. 2005). A Se–As–glutathione complex, seleno-bis(*S*-glutathionyl) arsinium ion, [(GS)_2_AsSe]^−^, has been detected in biliary excretion of rabbits, suggesting that one potential mechanism whereby Se intake may reduce the body burden of As is by increasing its loss through biliary excretion (Gailer et al. 2000), although this complex has not yet been definitively identified in humans. In this cross-sectional analysis, it is not possible to determine the temporal nature of the relationship between plasma Se and uAs/bAs or whether this is a causal relationship. An additional possibility is that As in the environment may adversely affect Se nutritional status. For example, a recent study reported that high concentrations of As in rice in Bangladesh are associated with lower levels of Se and other trace minerals in rice (Williams et al. 2009).

In the present study, we observed a significant inverse association between plasma Se and uAs, a finding that is in agreement with the results from a previous case–cohort study from our study area where higher blood Se concentrations were associated with lower uAs and reduced risk for As-induced premalignant skin lesions (Chen et al. 2007). In the present study, we observed an inverse association between plasma Se and bAs concentrations as well, despite the fact that we previously detected no association between whole-blood Se and bAs concentrations (Chen et al. 2007). The explanation for these discrepant findings is unclear. One difference between the studies is that we measured plasma Se in the present study, as opposed to whole-blood Se in the previous study.

### Plasma Se and As metabolites

Arsenic methylation and the toxicity of its metabolites have undergone considerable investigation in recent years. Compelling evidence from experimental data suggests that trivalent arsenical intermediates, in particular MMA^III^, are more toxic than their pentavalent counterparts (Petrick et al. 2000, 2001; Styblo et al. 2000). In agreement with the experimental data, population-based studies indicate that individuals with lower relative proportions of urinary DMA (and higher MMA) exhibit a greater risk for skin cancers (Chen et al. 2003; Hsueh et al. 1997; Yu et al. 2000) and bladder cancers (Huang et al. 2008), as well as peripheral vascular disease (Tseng et al. 2005). These results suggest that individuals who have a reduced capacity to fully methylate InAs to DMA^V^, the less toxic metabolite, are at heightened risk for As-induced health outcomes.

Antagonism between As and Se whereby each reduces the toxicity of the other has long been documented (Levander 1977; Moxon 1938). It is possible that As and Se may cause alterations in the biotransformation, distribution, and excretion of each other (Csanaky and Gregus 2003; Kenyon et al. 1997). For example, mice fed diets with excess Se^IV^ excreted significantly higher proportions of InAs than of methylated arsenicals in urine compared with mice fed Se-adequate diets (Kenyon et al. 1997). Experimental studies have shown that Se^IV^ exposure reduced the methylation of InAs in rat cytosol (Styblo et al. 1996). In a subsequent study, those authors also found Se^IV^ to be a potent inhibitor of recombinant arsenic methyltransferase (Walton et al. 2003). Furthermore, experiments in rat hepatocytes revealed that exposure to both Se^IV^ and As^III^ decreased the ratio of DMA to MMA, suggesting that the second As methylation step is more sensitive to Se than the first (Styblo and Thomas 2001). Conversely, in rats injected with either As^V^ or As^III^, Se^IV^ (10 μmol/kg, intravenous) lowered tissue concentrations of MMA^III^ and MMA^V^ but increased the concentration of DMA^V^ (Csanaky and Gregus 2003). However, the effect of supplementation with selenomethionine (commonly found in food and commercial Se supplements) on As methylation has not been tested, and the direct relevance of these studies employing inorganic forms of Se to the metabolism and toxicity of As in humans is unclear.

In the present study, we observed significant associations between plasma Se and proportions of As metabolites in blood. Plasma Se was associated with lower blood %MMA and higher blood %DMA. We observed no significant associations between Se and the individual proportions of As metabolites in urine. These results are not consistent with those of the animal studies. This may be due to the use of inorganic forms of Se in the animal studies and/or to important differences in As metabolism across species (Drobna et al. 2010). A potential mechanism by which Se may influence As metabolism is via the Trx/TR system. Trx can provide reducing equivalents for the reduction of MMA^V^ to MMA^III^, a prerequisite for the generation of DMA^V^. Se deficiency results in decreased activity of TR, a selenoprotein, thereby limiting the regeneration of Trx (Hill et al. 1997). Our data are consistent with the possibility that Se sufficiency may be permissive for the reduction of MMA^V^ to MMA^III^, consequently facilitating the methylation of MMA^III^ to DMA^V^.

## Conclusion

Our results indicate that plasma Se concentrations are inversely related to total bAs and uAs concentrations, inversely related to %MMA in blood, and positively associated with %DMA in blood. Collectively, the data suggest that Se may reduce the body burden of As and, moreover, may help to reduce concentrations of blood MMA, the most toxic metabolite in the As methylation pathway. The cross- sectional design of this study limits our ability to determine the temporal nature of our observed associations. Ongoing studies in Bangladesh are exploring the efficacy of Se supplementation in reducing As-induced health outcomes. The underlying mechanisms and biological implications of the inverse association between Se and genomic DNA methylation are unclear and warrant further investigation.

## Figures and Tables

**Table 1:** General characteristics of the study sample (*n* = 287).

Variable	Mean ± SD (range) or percent
Age (years)	37.9 ± 10.4 (18–66)
Male	48
BMI (kg/m^2^)	19.8 ± 3.2 (13.3–30.5)
Ever smoking	37.0
Ever betel nut use	33.8
Education (years)	3.3 ± 3.5 (0–16)
Type of housing	
Thatched	3.1
Corrugated tin	88.5
Other	8.4
Plasma measures	
Plasma Se (μg/L)	87.6 ± 1.8 (45.4–148.8)
Se deficient^a^	16
Plasma B_12_ (pmol/L)	276.3 ± 115.1 (86.1–920.0)
B_12_ deficient^b^	23
Plasma folate (nmol/L)	8.8 ± 4.3 (3.0–44.7)
Folate deficient^c^	63
Plasma homocysteine (μmol/L)	10.9 ± 5.2 (0.24–49.8)
Water As (μg/L)	113.6 ± 108.0 (0.1–716)
Urinary measures	
uAs (μg/L)	172.6 ± 172.4 (8.0–1519.0)
Urinary Cre (mg/dL)	60.5 ± 43.6 (5.6–334.8)
uAs/g Cre	339.0 ± 302.2 (21.0–2018.0)
%InAs	15.1 ± 6.1 (6.0–60.1)
%MMA	12.6 ± 4.2 (3.8–26.9)
%DMA	72.4 ± 7.8 (36.2–87.9)
Blood measures^d^	
bAs (μg/L)	9.9 ± 6.3 (0.89–30.7)
%InAs	26.3 ± 3.9 (16.2–40.2)
%MMA	40.3 ± 6.3 (15.9–68.5)
%DMA	33.4 ± 6.5 (7.6–48.0)
[^3^H]Methyl incorp	42,270 ± 3,984 (27,256–56,940)

Abbreviations: Cre, creatinine; incorp, incorporation.

a Plasma Se < 70.0 μg/L.

b Plasma cobalamin < 185 pmol/L.

c Plasma folate < 9 nmol/L.

d *n* = 223.

**Table 2:** Estimated parameters and 95% CIs for associations between increasing plasma Se (μg/L) and [^3^H]methyl incorporation, uAs, and bAs (*n* = 287).

	Unadjusted covariate		Adjusted covariate	
Outcome variable	Coefficient estimate (95% CI)	*p*-Value^a^	Coefficient estimate (95% CI)^b^	*p*-Value^a^
[^3^H]Methyl incorporation	395.5 (135.0 to 656.1)	0.003	345.6 (59.1 to 632.2)	0.02
Urinary measures				
Total uAs	−16.6 (−27.9 to −5.3)	0.004	−20.1 (−29.3 to −10.9)	< 0.0001
Urinary percent InAs	−0.28 (−0.68 to 0.12)	0.17	0.07 (−0.33 to 0.46)	0.74
Urinary percent MMA	0.20 (−0.08 to 0.48)	0.16	0.18 (−0.10 to 0.46)	0.21
Urinary percent DMA	0.08 (−0.44 to 0.60)	0.76	−0.25 (−0.77 to 0.28)	0.36
Blood measures^c^				
Log total bAs (μg/L)	−0.05 (−0.10 to −0.01)	0.03	−0.04 (−0.08 to −0.01)	0.03
Blood percent InAs	0.07 (−0.22 to 0.36)	0.66	0.06 (−0.24 to 0.36)	0.69
Blood percent MMA	−0.57 (−1.05 to −0.10)	0.02	−0.59 (−1.04 to −0.13)	0.01
Blood percent DMA	0.51 (0.02 to 1.00)	0.04	0.53 (0.04 to 1.01)	0.03

a *p*-Value from a test of the null hypothesis of coefficient equal to zero.

b Adjusted for age, sex, BMI, ever smoking, ever betel nut use, plasma folate (log), plasma B_12_ (log), urinary creatinine (log), and water As (square root).

c *n* = 223. Adjusted for age, sex, BMI, ever smoking, ever betel nut use, plasma folate (log), plasma B_12_ (log), and water As (square root).

**Table 3:** Adjusted^a^ mean (95% CI) of outcome variables by plasma Se category (*n* = 287).

	Se category [μg/L; mean (range)]				
	1	2	3	4	5
Outcome variable	4.54–7.01 (*n* = 44)	7.02–8.15 (*n* = 60)	8.16–8.94 (*n* = 61)	8.95–10.03 (*n* = 61)	10.04–14.88 (*n* = 61)
[^3^H]Methyl incorporation (dpm/μg DNA)	41,053 (38,909–42,296)	42,273 (41,223–43,324)	42,125 (41,120–43,130)	42,672 (41,641–43,703)	42,988 (41,937–44,039)
Total uAs (μg/L)	221.1 (181.2–261.0)	191.7 (158.0–225.4)	188.6 (156.4–220.8)	158.6 (125.5–191.7)	120.3 (86.6–154.0)
Total bAs (μg/L)^b^,^c^,^d^	8.9 (7.7–10.3)	8.3 (7.3–9.5)	8.8 (7.6–10.2)	7.8 (6.8–9.1)	7.4 (6.4–8.6)
Blood %MMA^b^,^d^	41.6 (39.8–43.3)	40.9 (39.3–42.5)	41.5 (39.7–43.2)	38.4 (36.7–40.2)	39.6 (37.8–41.4)
Blood %DMA^b^,^d^	32.0 (30.1–33.9)	33.3 (31.5–35.0)	32.6 (30.7–34.5)	34.4 (42.5–36.3)	34.5 (32.5–36.4)

Category 1, Se deficient participants; categories 2–5, quartiles of Se sufficient participants.

a Adjusted for age, sex, BMI, ever smoking, ever betel nut use, plasma folate (log), plasma cobalamin (log), urinary creatinine (log), and water As (square root).

b *n* = 223.

c Values are geometric means.

d Adjusted for age, sex, BMI, ever smoking, ever betel nut use, plasma folate (log), plasma cobalamin (log), and water As (square root).
